# Yet another Bradford's law: new evidence on integrated care from Japan

**DOI:** 10.5334/ijic.1588

**Published:** 2014-06-23

**Authors:** Etsuji Okamoto

**Affiliations:** National Institute of Public Health, Japan

In this edition of *IJIC*, a bibliometric study of academic journals in the field of integrated care quoted the “Bradford's law”: a skewed concentration of academic articles in “core” journals [1]. The article also demonstrated yet another Bradford's law: a skewed concentration of articles in a small number of countries with little relevance to population size. In particular, the distribution of evidence is heavily skewed towards articles from the USA and Northern Europe. In contrast, China and India, each occupying 19.5% and 17.8% of the world population, represent only 0.2% and 0.4% of publications, respectively, despite their recent focus on policies for developing primary and community-based integrated care.

The law also seems to apply to both economic size and countries with social insurance systems. For example, Japan's Long-term Care Insurance (LTCI) is unquestionably one of the largest in the world in economic size: insuring 30 million elderly and providing care to 5 million current recipients with an annual budget of 10 trillion yen (approximately 100 billion dollars), and Japan has also developed innovations in care pathways and case management. However, articles from Japan accounted for a pitiful 0.32%. If this law holds true, articles from under-represented countries might be viewed as “a few but significant”, so the recent articles from Japan published in *IJIC* during 2014 provide the much-needed emphasis on a country with an interesting history in this area.

Japan's LTCI was introduced in 2000 after a lengthy policy debate and technical preparation, starting in 1995. One of the hotly debated issues at that time was whether cash benefit be granted for family caregivers. In view of the potential moral hazard, the cash benefit was not included, unlike the preceding German LTCI. The resulting Japan's LTCI was characterized by two innovations: introduction of an official care management system and a need assessment tool to certify eligibility [2], the content of which was described by Matsuda et al. in the inaugural issue of *IJIC* [3].

After 14 years since its inception, the LTCI has taken firm root in Japan's society. Consequently, the financial size of the LTCI grew rapidly. The number of service recipients more than tripled from 1.5 million in 2000 to 5.3 million in 2012, and the annual budget increased from 3.6 trillion yen to projected 10 trillion yen in 2014. Inevitably, the focus of the LTCI shifted from expansion to restructuring.

Two recent policy papers in *IJIC* describe the restructuring process under way in Japan. Morikawa describes problems of care management as “structural weakness” of Japan's LTCI [4]: there is no clear division between care management and service provision, which leaves it open to abuse by care managers employed by profit-seeking private organizations. This explains why the Japanese community generally has support centres that have been set up in each municipality to prevent service abuses as part of the 2005 reform.

Tsutsui, who was also involved in the development of the need assessment tool in the early phase of Japan's LTCI [5], advocates the future vision dubbed “community-based integrated care system” for which she contributed as a member of the Research Committee [6]. The key idea of this vision is “housing (residential care)”. With proper housing and home care services, one can reduce costly institutional care, thereby contributing to “sustainability of the funding system”.

A further research and theory paper by Taguchi et al. proposes an assessment tool to measure the need for nursing services [7]. Before the introduction of LTCI, nursing services were funded by health insurance and social services through government subsidy. The creation of the LTCI, however, provided a rivalry between health and social care jurisdictions over the limited budget allocated to each by care needs. The authors might have been alerted at the disadvantageous position of nurses in Japan: “the use of home visiting nursing service in the LTCI in 2010 increased only 130% since 2001, whereas the use of other services increased by 170%”. The authors’ concern echoes the problems of care management raised by Morikawa. If care managers are motivated solely by profit or financial considerations they may not make the right decision. The assessment tool by the authors, therefore, may provide a solution but needs to consider the complex financial and policy environment in which nurses operate.

Tsutsui concludes that “in Asian countries, where the ageing is even more sudden than Japan, it is expected that integrated care system will soon be needed”; then one might expect a sharp increase of articles from the hitherto under-represented countries in the literature as a paradigm shift towards integrated systems of care takes place.
